# Dental care for older adults in home health care services - practices, perceived knowledge and challenges among Norwegian dentists and dental hygienists

**DOI:** 10.1186/s12903-023-02951-x

**Published:** 2023-04-18

**Authors:** Marte-Mari Uhlen-Strand, Ewa Alicja Szyszko Hovden, Falk Schwendicke, Vibeke Elise Ansteinsson, Ibrahimu Mdala, Rasa Skudutyte-Rysstad

**Affiliations:** 1 Oral Health Centre of Expertise in Eastern Norway (OHCE-E), Oslo, Norway; 2 Charité – Universitätsmedizin Berlin, Berlin, Germany; 3 Department of Public Health Science, Faculty of Medicine, Institute of Health and Society, University of Oslo, Oslo, Norway

**Keywords:** Oral health, Older adults, Home health care, Dental care

## Abstract

**Background:**

Providing dental services to dependent older adults might be challenging because of physical and cognitive decline. The present study aimed to explore current practices, knowledge, and experienced challenges related to the treatment of older adults in home health care services (HHCS) among dentists and dental hygienists in Norway.

**Methods:**

An electronic questionnaire survey was distributed to Norwegian dentists and dental hygienists, inquiring about background characteristics, current practices, self-perceived knowledge, and challenges when providing oral health care for older HHCS patients.

**Results:**

Four hundred and sixty-six dentists and 244 dental hygienists treating older HHCS patients responded to the survey. The majority were female (*n*=620; 87.3%) and worked in the public dental service (PDS) (*n*=639; 90%). When older HHCS adults attended the dental practice, the treatments provided were most frequently aimed at relieving acute oral problems, although dental hygienists reported to focus on improving oral health more often than dentists. Dentists reported to have more self-perceived knowledge than dental hygienists regarding patients with complex treatment needs, cognitive or physical impairment. Exploratory Factor Analysis (EFA) was carried out on the 16 items related to challenges, three factors were extracted and Structural Equation Models (SEMs) were performed. Challenges related to dental care for older HHCS adults were related to time, practical organization and communication. Variation within these categories was associated with sex, graduation year and country, as well as time used per patient and work sector, but not with professional status.

**Conclusions:**

The results indicate that dental care for older HHCS patients is time-demanding and more often aimed at relieving symptoms than improving oral health. A substantial proportion of dentists and dental hygienists in Norway lack confidence when providing dental care for frail elderly.

**Supplementary Information:**

The online version contains supplementary material available at 10.1186/s12903-023-02951-x.

## Introduction

The proportion of the elderly retaining their own teeth is increasing, and epidemiological evidence suggests that the burden of caries and periodontal diseases is expected to grow in aging populations, including frail elderly [1]. Maintaining good oral health is based on adequate oral hygiene and regular access to dental services, with oral health being an important determinant of overall health and wellbeing [2, 3]. Risk factors for oral diseases accumulate throughout life, and the majority of older people will therefore continue to have a need for both preventive and curative oral health care [4, 5].

Becoming older is associated with a higher incidence of illnesses and conditions that may lead to care dependency and increased vulnerability [6, 7]. However, caring for patients in their homes is becoming the preferred mode of health care delivery among the elderly population [8, 9].

Several studies have shown that oral health among frail and dependent elderly is inferior to the general population [10]. When physical and cognitive functions are impaired, the capacity to perform oral hygiene is often reduced, thus older people may become dependent on assistance with daily oral care [11, 12]. Moreover, older people in Western countries use dental health care services far less often than younger people [4], and frail people visit dental clinics less frequently compared to non-frail people [13]. The frequency of dental visits and the relative proportions of diagnostics and prevention decrease with age, with older patients mainly visiting their dentist for restorative or prosthetic care [2, 14].

As preventive measures and treatment strategies for oral diseases are effective at all ages, the same standard of prevention and care should be provided across the entire life span [15]. Providing dental services to dependent older people might be challenging due to reduced mobility, physical and cognitive decline, multimorbidity, and polypharmacy [16]. Previous studies have assessed and identified barriers for continued dental service provision for the care-dependent elderly and have pointed out the lack of suitable facilities, transportation, refusal of care, as well as the lack of adequate training and experience among clinicians [4, 11].

Elderly in home care have been found to have poorer oral health than nursing home residents [3]. Most international studies on the oral health of people in need of long-term care have focused primarily on the nursing home setting, and less is known about the provision of dental care to the elderly in domiciliary care [3, 4]. In addition, most studies exploring the dentists’ experience in delivering oral health care to older people are surveys focusing on oral health care in nursing homes. It has been shown that frail older people newly admitted to nursing homes often have poor oral health, which indicates that the deterioration has already taken place [12]. More focus on the prevention of oral diseases and interventions to improve oral health and dental care among home care recipients is therefore needed. More research should be carried out to investigate the current practices for dental care and preventive advice for older adults in home health care services (HHCS), as well as the challenges dental health personnel experience while delivering oral health care to this patient group [3, 4].

According to Statistics Norway, there were 4919 dentists and 1153 dental hygienists registered in Norway in 2021. For a population of 5.4 million, the number of inhabitants per dentist is approximately 1100, which indicates relatively good access to dental care [17]. Care-dependent elderly are entitled to free dental care in the Public Dental Service (PDS), however, only approximately 20% of those in HHCS are using PDS [18]. The reasons for this have not been fully investigated. We lack knowledge about preventive practices as well as treatment procedures that dental professionals provide for HHCS patients. Moreover, dentists’ and dental hygienists’ experiences and challenges related to providing dental services for care-dependent older adults have not been explored.

Thus, the aim of the present study was to explore current practices, knowledge, and experienced challenges related to the treatment of older HHCS adults among dentists and dental hygienists in Norway.

## Methods

### Study design and participants

This was an explorative nationwide survey among dentists and dental hygienists in Norway. For the PDS, the chief dental officers in all counties in Norway were contacted and asked to distribute the questionnaire by e-mail among clinicians working in public dental clinics. Invitations to dental hygienists were sent by e-mail via the Norwegian Dental Hygienist Association. Invitations to dentists in the private sector were distributed via newsletters and social media to members of the Norwegian Dental Association. Data collection was based on an electronical questionnaire distributed via QuestBack and started on 15 November 2021. Three reminders for participation were sent, and the data collection ended on 16 January 2022. Only dentists and dental hygienists who reported to provide dental care for older HHCS adults (65+) were included.

### Ethics approval and consent to participate

Participation was voluntary and written informed consent was obtained electronically from all participants. No compensation was given to the respondents. Anonymity of the respondents was ensured by QuestBack. The study was approved by the Norwegian Centre for Research Data (210679). All methods were performed in accordance with relevant guidelines and regulations. The manuscript was prepared according to STROBE guidelines [19].

### Variables

The questionnaire consisted of four parts:

1. Background characteristics (Table 1)

**Table 1:** Background characteristics of dentist and dental hygienists working with older patients (+65) in HHCS

**Background characteristics**	**Dentist (% within column) *n*=466**	**Dental hygienist (% within column) *n*=244**	**Total *n*=710**	***P*-value**
**Gender**				**<0.001**
**Female**	383 (82.2)	237 (97.1)	620 (87.3)	
**Male**	83 (17.8)	7 (2.9)	90 (12.7)	
**Age**				**<0.001**
**≤ 30**	85 (18.2)	41 (16.8)	126 (17.7)	
**31 – 40**	189 (40.6)	53 (21.7)	242 (34.1)	
**41 – 55**	144 (30.9)	105 (43.0)	249 (35.1)	
**56+**	48 (10.3)	45 (18.4)	93 (13.1)	
**Graduation year**				**<0.001**
**≤ 2007**	197 (42.4)	149 (61.1)	346 (48.8)	
**2008 - 2021**	268 (57.6)	95 (38.9)	363 (51.2)	
**Graduation country**				**<0.001**
**Norway**	345 (74.0)	236 (96.7)	581 (81.8)	
**Other**	121 (26.0)	8 (3.3)	129 (18.2)	
**Sector**				**0.016**
**Public**	426 (91.4)	213 (87.3)	639 (90.0)	
**Private**	18 (3.9)	22 (9.0)	40 (5.6)	
**Combined**	22 (4.7)	9 (3.7)	31 (4.4)	
**Part-time or full-time position**				**0.025**
**Part-time**	72 (15.5)	54 (22.2)	126 (17.8)	
**Full-time**	394 (84.5)	189 (77.8)	583 (82.2)	
**Average number of older adults in HHCS patients treated per week**				**<0.001**
**4 or less**	244 (52.4)	161 (66.0)	405 (57.0)	
**5 or more**	222 (47.6)	83 (34.0)	305 (43.0)	
**Average time used per appointment**				**<0.001**
**Less than 45 minutes**	64 (13.8)	62 (25.6)	126 (17.8)	
**45 minutes or more**	401 (86.2)	180 (74.4)	581 (82.2)
2. Current practices (dental care and preventive advice)
  Dentists were presented 12 dental procedures and asked to rank up to four of their most common procedures when providing dental care for older HHCS adults (Figure 1).

**Fig. 1:**
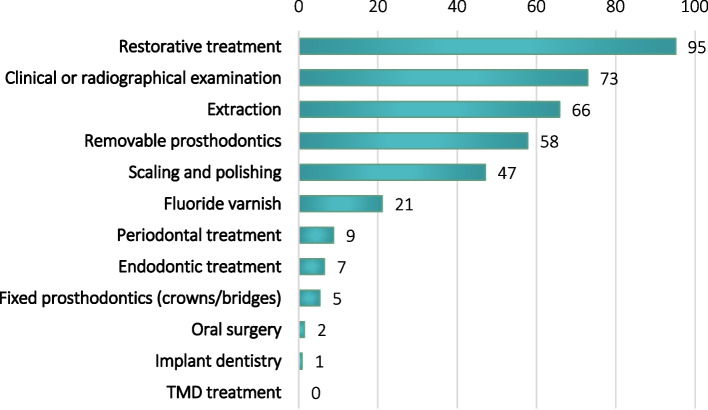
Most common dental procedures reported by dentists (%). TMD – Temporomandibular disorders
  Dentists and dental hygienists were asked how often they gave preventive advice concerning brushing technique, interdental cleaning, use of fluorides at home and diet on a 5-point Likert scale (always, often, sometimes, seldom, never) (Figure 2).

**Fig. 2:**
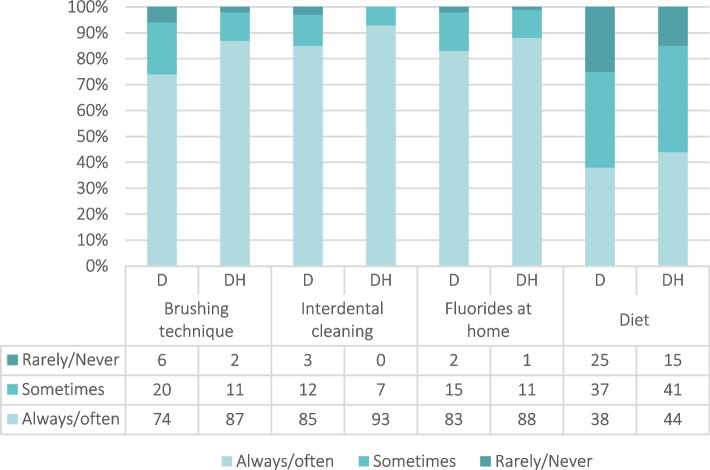
Frequency of preventive advices given by dentists (D) and dental hygienists (DH) to older adults in HHCS by occupation (%)
  Dentists and dental hygienists were asked to state if the treatments they provided were most often performed to relieve oral problems/symptoms, postpone, preserve, or improve the patient’s oral health status.
3. Self-perceived knowledge
  The respondents were asked to evaluate their self-perceived knowledge regarding treatment of older patients within three different categories: Patients with complex treatment needs, patients with dementia or other cognitive impairment, and patients with impaired physical functioning. The responses were given on a 5-point Likert scale (totally agree, agree, neither agree nor disagree, disagree, totally disagree) (Table 2).

**Table 2:** Self-perceived knowledge about dental treatment of older patients (+65) in HHCS

**I have enough knowledge about dental treatment of older patients (65+) in HHCS…**	**Dentists**		**Dental** **hygienists**		**Total**		***p***
	**n**	**%**	**n**	**%**	**n**	**%**	
**…with complex treatment needs**							
Totally agree/agree	258	55.5	92	37.9	350	49.4	**<0.001**
Neither agree nor disagree	154	33.1	94	38.7	248	35.0	**<0.001**
Disagree/totally disagree	53	11.4	57	23.5	110	15.5	**<0.001**
**…with dementia or cognitive impairment**							
Totally agree/agree	235	50.5	96	39.5	331	46.8	**<0.001**
Neither agree nor disagree	167	35.9	85	35.0	252	35.6	**<0.001**
Disagree/totally disagree	63	13.5	62	25.5	125	17.7	**<0.001**
**…with impaired physical functioning**							
Totally agree/agree	280	60.2	114	46.9	394	55.6	**<0.001**
Neither agree nor disagree	145	31.2	86	35.4	231	32.6	**<0.001**
Disagree/totally disagree	40	8.6	43	17.7	83	11.7	**<0.001**
4. Experienced treatment-related challenges
  Dentists and dental hygienists were presented 16 statements about challenging situations relevant for dental treatment of older patients and asked to report how often they experienced each of them, on a 5-point Likert scale (always, often, sometimes, seldom, never) (Figure 3).

**Fig. 3:**
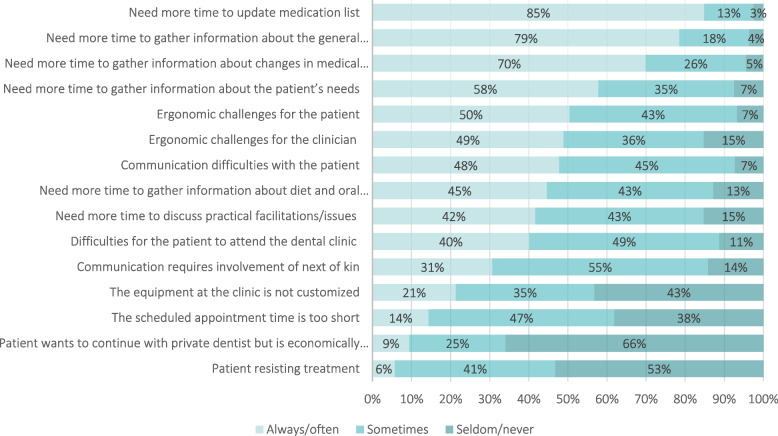
Experiences and perceived challenges of dentists and dental hygienists when providing oral health care for older patients in HHCS (%)

The questionnaire was based on the topics from the literature and pilot-tested for face-validity by ten dentists and dental hygienists in public and private dental service to ensure respondents comprehension of the questions and length of the questionnaire. Content validity was also assessed with the same clinicians to determine whether the questionnaire captured the intended research objectives.

### Statistical methods

Descriptive statistics in the form of frequency and percentage distributions were used to describe the background characteristics of the respondents. Chi-squared test was used to test bivariate associations.

From the 16 items on experienced challenges, we conducted an exploratory factor analysis (EFA) using oblique rotation in order to identify and examine clusters of inter-correlated variables. We extracted three clusters of variables called factors, which explained most of the observed variance of the original 16 items. The sampling adequacy of the data for EFA was assessed using the Kaiser-Meyer-Olkin (KMO) statistic, which was considered to be meritorious (KMO = 0.859). In constructing the factors, all items exhibiting factor loads of ≥ 0.30 were considered.

Structural equation models (SEMs) were then used to identify socio-demographic characteristics that were significantly associated with the factors extracted.

IBM SPSS Statistics 27 was used to perform descriptive analyses whereas Stata SE 17 was used for conducting EFA and SEMs. The level of statistical significance was set at 5%.

## Results

The background characteristics are presented in Table 1. The vast majority of the respondents were female, graduated in Norway, worked in the PDS and had a full-time position. All background characteristics that were recorded differed significantly between dentists and dental hygienists. Dental hygienists were generally older than dentists, had graduated before 2008 and were more often educated in Norway. In addition, there was a larger proportion of dental hygienists working in the private dental service and a larger proportion of dental hygienists with part-time positions.

### Treatment practices and self-perceived knowledge

Of the respondents, 18% reported that older HHCS adults came to the dental clinic because of pain or acute problems (20% of dentists, 14% of hygienists, *p*<0.025). According to the respondents, treatment was most often aimed at relieving oral problems (49%), preserving (28%) and improving oral health (24%). Dental hygienists were more often focused on improving oral health compared to dentists (27% versus 23%, *p*=0.009).

The most frequent procedures performed by dentists are shown in Figure 1. Restorative treatments (fillings) were most frequently performed, followed by clinical and radiographical examinations and extractions. Less than 10% reported that periodontal treatment, endodontic treatment or fixed prosthetic treatment was among their most frequently performed procedures, and less than 3% frequently performed oral surgery, implant dentistry or temporomandibular joint treatment on this patient group.

Figure 2 shows the frequency of preventive advice given by clinicians to older HHCS adults. The majority of respondents reported to always/often give preventive advice about brushing technique, interdental cleaning and use of fluorides at home, but less than half reported to always/often give dietary advice. A larger proportion of dental hygienists than dentists reported to give preventive advice in all four categories.

There were statistically significant differences between dentists and dental hygienists considering their self-perceived knowledge regarding patients with complex treatment needs, cognitive or physical impairment (*p*<0.001) (Table 2). Slightly more than half of the dentists totally agreed or agreed that they had enough knowledge in all three areas (50.5%, 60.2% and 55.5%, respectively), compared to less than half of the dental hygienists (39.5%, 46.9% and 37.9%, respectively).

### Experienced challenges

The vast majority of dentists and hygienists reported that they always/often had to use extra time to update the older HHCS patients’ medication lists and patient history (Figure 3). On the other hand, very few reported experiencing patients who resisted treatment or patients who wanted to continue in the private dental practice but were hindered by economic reasons. Seven of the 16 statements revealed statistically significant different experiences between dentists and dental hygienists (Table S1).

Using EFA, three factors with eigenvalues above the Kaiser’s criterion of 1 were extracted and explained 51.92% of the variance. Table 3 shows the factor loadings ≥ 0.30. Six items clustered on Factor 1, which represents challenges related to time needed to gather essential information of the patients (Time); another six items clustered on Factor 2 representing challenges related to resources and practical issues (Practical organization), while three items clustered on Factor 3 representing communication problems (Communication). Factor 1 had high reliability, while Factor 2 and 3 had moderately high reliabilities.

**Table 3:** Summary of the FA results showing factor loadings ≥ 0.30 on experiences and perceived challenges when providing dental care of older people in HHCS

**Item**	**Factor loadings ≥ 0.30**		
	**Factor 1** **Time**	**Factor 2** **Practical organization**	**Factor 3** **Communication**
**Need for more time to gather information about changes in the patient’s medical condition**	0.876		
**Need for more time to gather information about the general anamnesis of the patient**	0.827		
**Need for more time to gather information about the patient’s needs**	0.757		
**Need for more time to update the patient’s medication list**	0.738		
**Need for more time to gather information about the diet and oral hygiene habits of the patient**	0.505		
**Need for more time to discuss practical facilitations/issues**	0.502		
**The equipment at the clinic is not adapted for patients with impaired functioning**		0.622	
**The scheduled appointment time is too short**		0.547	
**Ergonomic challenges for the patient make the treatment difficult**		0.495	
**Ergonomic challenges for the clinician make the treatment difficult**		0.471	
**The patient wants to continue in the private dental service, but is hindered by economic reasons**		0.370	
**Difficulties for the patient to attend the dental clinic**		0.302	
**Communication difficulties with the patient due to dementia, aphasia, hearing problems etc**			-0.648
**Patient resisting treatment**			-0,616
**Communication concerning dental treatment requires involvement of next of kin (patient’s relatives)**			-0.561
Eigenvalues	4.533	2.048	1.201
% of variance	30.26	13.66	8.01
Cronbach’s alpha: overall = 0.832	0.855	0.674	0.644

Standardized coefficients obtained from SEMs showed that males were more likely than females to consider practical organization to be a challenge when providing dental treatment to older HHCS patients (Table 4). Dentists and dental hygienists who graduated in 2008 or later were less likely to experience challenges with the time needed to gather essential information about the patient and practical organization. However, they were more likely to experience communication problems than those who graduated before 2008. Clinicians who graduated in countries other than Norway were less likely to report problems regarding time. Those who reported using 45 minutes or more per patient were less likely to experience challenges with time and practical concerns than those using less time per patient. Clinicians working in the private dental service were more likely to experience practical challenges and were less likely to experience communication problems than those working in the PDS.

**Table 4:** Standardized coefficients obtained from Structural Equation Models (SEMs). Significant associations are showed in bold

**Characteristics**	**Factor 1** **Time**		**Factor 2** **Practical organization**		**Factor 3** **Communication**	
	β (95% CI)	*P*-value	β (95% CI)	*P*-value	β (95% CI)	*P*-value
**Occupation (ref: Dentist)**						
**Dental hygienist**	0.01 (-0.08, 0.09)	0.88	-0.07 (-0.15, 0.01)	0.08	-0.01 (-0.09, 0.07)	0.87
**Gender (ref: Female)**						
**Male**	0.01(-0.06, 0.09)	0.74	**0.08 (0.005, 0.15)**	**0.04**	-0.07 (-0.14, 0.01)	0.08
**Graduation year (ref: ≤ 2007)**						
** (2008 – 2021)**	**-0.14 (-0.21, -0.06)**	**< 0.01**	**-0.23 (-0.30, -0.15)**	**< 0.01**	**0.13 (0.05, 0.20)**	**< 0.01**
**Graduation country (ref: Norway)**						
**Other**	**-0.08 (-0.16, -0.004)**	**0.04**	-0.05 (-0.13, 0.03)	0.20	0.06 (-0.02, 0.13)	0.15
**Time used per patient** **(ref: < 45 minutes)**						
**≥ 45 minutes**	**-0.08 (-0.15, -0.001)**	**0.05**	**-0.08 (-0.16, -0.01)**	**0.03**	0.03 (-0.05, 0.10)	0.49
**Sector (ref: Public)**						
**Private**	-0.03 (-0.10, 0.05)	0.51	**0.08 (0.01, 0.16)**	**0.03**	**-0.22 (-0.29, -0.15)**	**< 0.01**
**Vacancy rate (ref: Part-time)**						
**Full-time**	-0.05 (-0.12, 0.03)	0.20	-0.01 (-0.08, 0.07)	0.85	0.07 (-0.01, 0.14)	0.08
**Number of patients (ref: ≤ 4)**						
**≥ 5**	-0.05 (-0.12, 0.03)	0.22	-0.05 (-0.13, 0.02)	0.17	0.06 (-0.01, 0.13)	0.09

## Discussion

In the present study, the current practices, self-perceived knowledge, and experienced challenges of Norwegian dentists and dental hygienists when providing dental care to older HHCS adults were explored. Our results demonstrated that dental care for this population was mainly aimed at relieving acute problems rather than preserving or improving patients’ oral health. This is in accordance with the most frequent reported treatments offered by dentists which, with the exception of clinical and radiographical examinations, were fillings, extractions, and removable prostheses. In contrast, fluoride varnish application, periodontal and endodontic treatment were performed less frequently. This is in agreement with previous studies showing that restorative treatments were the dominating treatment procedures given by dentists to older adults in Western countries [20], and that periodontal treatment was seldom performed [21].

Studies investigating the oral health of older patients have shown that the proportion of older adults retaining their natural teeth in the general population is increasing [22, 23], however, the prevalence of edentulism among HHCS elderly is still high [10]. In the present study, 95% of the dentists reported fillings to be one of their most frequently performed procedures, indicating that a substantial proportion of this population are dentate.

Dentists were more likely to experience patients contacting them due to pain compared to dental hygienists (20% versus 14%). This is not unexpected, considering the different work responsibilities of dentists and dental hygienists. As the frequency of dental visits has been shown to decline with age and worsening general health [13], it is reasonable to believe that the institutionalized elderly visit the dental clinic less often than those living at home [24]. It has also been reported that older people who visit community dental practices are still relatively healthy, non-frail, and highly educated [16].

In Norway, only a small proportion of older adults in HHCS use their rights to free dental care, and the reason for this is not known. However, a questionnaire study among HHCS users revealed that only 49% were aware of the possibility of free dental care [25]. It has been speculated that not all eligible older adults may receive the information about free dental treatment or that some may simply forget receiving the information [25]. In other countries, factors that influence the dental service utilization in this patient group have been identified, such as the presence of pain, need for prosthetic treatment, education level, or financial situation. Lack of suitable facilities for transportation or treatment, staff and time constraints, as well as lack of awareness and knowledge among nursing staff have been reported as barriers. In addition, individuals without any or with only functional limitations and better mental health tended to use dental services more frequently [11, 13, 24].

Poor oral hygiene, a sugar-rich diet, and dry mouth are common risk factors for oral diseases [5]. Earlier studies have pointed to a lack of awareness regarding oral health and hygiene among healthcare providers, patients, and relatives – resulting in poor oral health [26]. Substantial treatment needs as well as inadequate daily oral hygiene have been revealed among older adults in HHCS [10]. In addition to the increased emphasis on the assistance with daily oral care provided by caregivers in HHCS, it is important that dental health personnel focus on preventive measures when meeting patients and their caregivers. In the present study, both dentists and dental hygienists frequently recommended fluoride supplements for home use and gave advice on brushing technique and interdental cleaning. However, it is alarming that 25% of dentists and 15% of dental hygienists reported never giving dietary advice, and that less than half gave dietary advice often. Generally, dental hygienists provided more preventive services than dentists, which is in line with different professional responsibilities.

In the present study, between 47% and 56% of all clinicians reported having enough knowledge about dental treatment of patients with complex treatment needs, dementia or cognitive impairment, or with impaired physical functioning. Dentists were more confident than dental hygienists when treating these patients. Several studies have reported a demand for further training related to care of older people, especially when managing patients with dementia. Lack of knowledge, adequate training, and experience have been reported to be barriers when providing dental care to dependent older patients [4, 11].

Almost all respondents reported needing more time to gather essential information from patients. Limited resources and time have been described as barriers against providing the necessary dental care. In addition, physical limitations, multimorbidity and polypharmacy may lead to medical complexity and an unstable overall health, which may hamper the treatments [26]. The challenges experienced by dentists and dental hygienists in the present study were consolidated into three domains: time, practical organization, and communication. Respondents’ work experience showed an impact on all of the extracted factors from the EFA. Dentists and dental hygienists with more experience were more likely to experience challenges regarding time and practical organization than those with the shortest work experience. Almost all respondents highlighted the time efforts needed to update individual patients’ medication list or their general history, in line with previous evidence [11]. These findings indicate the need for increased focus on communication, information exchange, and interprofessional collaboration issues as users of HHCS have health issues and comorbidities which often require multidisciplinary conversation. In contrast to earlier studies that have found refusal of treatment and lack of suitable facilities as main barriers for dental treatment of dependent older people [11], however, very few respondents reported this to be a challenge in the present study.

In addition, suitable facilities for dental treatment or patient transportation have been found to be important for dentists to provide oral care for older patients [11]. This is in contrast with this study, where very few respondents reported a lack of customized equipment to be a challenge for dental treatment. Nevertheless, a large proportion reported ergonomic issues for the clinician or the patient as challenging.

This study comes with a number of limitations. This was a voluntary questionnaire study and study participants self-selected to complete the survey. Selection bias related to personal interests of respondents may have occurred. Furthermore, recall bias among participants as well as confirmation bias cannot be ruled out. Approximately 10% of the total number of registered dentists and 20% of dental hygienists in Norway responded to the questionnaire [17]. Public dentists, females and younger individuals were overrepresented among the respondents, thus, generalization of our findings should be made with caution.

To the best of our knowledge, the present study is the first in Norway to highlight the dentists’ and dental hygienists’ perspectives by investigating current practices, self-perceived knowledge and experienced challenges when providing dental care to older HHCS adults.

## Conclusion

In this study population, challenges related to dental care for older adults receiving HHCS could be categorized into time, practical organization and communication. Variation in perceived challenges was associated with background characteristics and sector, but not with the professional category of clinicians. The results indicated that dental care for older patients is time-demanding and more often aimed at relieving symptoms than preserving or improving the oral status. A substantial proportion of dentists and dental hygienists in Norway lacked confidence in providing dental care for older HHCS adults. Thus, more emphasis both in the undergraduate and postgraduate curricula of dental personnel is needed in order to meet the complex interprofessional needs for this increasing patient group.

## Supplementary Information

**Additional file 1: Table S1. **Experiences and perceived challenges among dentists and dental hygienists.

## Data Availability

The dataset used in the current study is available from the corresponding author on reasonable request.

## Abbreviations

EFA: Exploratory factor analysis
D: Dentists
DH: Dental hygienists
HHCS: Home health care service
KMO: Kaiser-Meyer-Olkin
PDS: Public dental service
SEM: Structural equation modeling
