# Supplementary material for: Dental care for older adults in home health care services - practices, perceived knowledge and challenges among Norwegian dentists and dental hygienists
Source: BMC Oral Health. 2023 Apr 18;23:222. doi: 10.1186/s12903-023-02951-x (PMC10111733; doi:10.1186/s12903-023-02951-x)
Supplement: Supplementary file 1 — Additional file 1: Table S1. Experiences and perceived challenges among dentists and dental hygienists. [file 12903_2023_2951_MOESM1_ESM.docx]

**Supplementary Table S1. Experiences and perceived challenges among dentists and dental hygienists**

| Item | Frequency of perceived challenges | | | | | |  |
| --- | --- | --- | --- | --- | --- | --- | --- |
|  | **Always/often** | | **Sometimes** | | **Seldom/never** | | ***p*** |
|  | **Dentists** | **Dental hygienists** | **Dentists** | **Dental hygienists** | **Dentists** | **Dental hygienists** |  |
| Need for more time to gather information about changes in the patient’s medical condition* | 42.4%  197 | 35.8%  87 | 46%  214 | 53.5%  130 | 11.6%  54 | 10.7%  26 | **0.008** |
| Need for more time to gather information about the general anamnesis of the patient* | 83.4%  388 | 69.1%  168 | 14.2%  66 | 24.7%  60 | 2.4%  11 | 6.2%  15 | **<0.001** |
| Need for more time to gather information about the patient’s needs | 59.8%  278 | 53.9%  131 | 32.9%  153 | 38.3%  93 | 7.3%  34 | 7.8%  19 | 0.312 |
| Need for more time to update the patient’s medication list* | 88.6%  412 | 77.8%  189 | 9.0%  42 | 19.3%  47 | 2.4%  11 | 2.9%  7 | **<0.001** |
| Need for more time to gather information about the diet and oral hygiene habits of the patient | 42.6%  198 | 48.6%  118 | 43.4%  202 | 40.7%  99 | 14.0%  65 | 10.7%  26 | 0.236 |
| Need for more time to discuss practical facilitations/issues | 43.0%  200 | 39.1%  95 | 41.7%  194 | 45.7%  111 | 15.3%  71 | 15.2%  37 | 0.558 |
| The equipment at the clinic is not customized | 21.5%  100 | 21.0%  51 | 36.3%  169 | 33.7%  82 | 42.2%  196 | 45.3%  110 | 0.711 |
| The scheduled appointment time is too short* | 12.7%  59 | 17.7%  43 | 46%  214 | 50.2%  122 | 41.3%  192 | 32.1%  78 | **0.032** |
| Ergonomic challenges for the patient* | 54.2%  252 | 43.2%  105 | 38.7%  180 | 50.6%  123 | 7.1%  33 | 6.2%  15 | **0.010** |
| Ergonomic challenges for the clinician * | 52.5%  244 | 42.0%  102 | 32.3%  150 | 42.8%  104 | 15.3%  71 | 15.2%  37 | **0.014** |
| The patient wants to continue in the private dental service, but is hindered * | 8.4%  39 | 11.5%  28 | 22.4%  104 | 28.8%  70 | 69.2%  322 | 59.7%  145 | **0.037** |
| Difficulties for the patient to attend the dental clinic | 42.4%  197 | 35.8%  87 | 46.0%  214 | 53.5%  130 | 11.6%  54 | 10.7%  26 | 0.160 |
| Communication difficulties with the patient | 49.9%  232 | 43.6%  106 | 43.7%  203 | 47.3%  115 | 6.5%  30 | 9.1%  22 | 0.196 |
| Patient resisting treatment | 4.9%  23 | 7.4%  18 | 41.1%  191 | 40.7%  99 | 54.0%  251 | 51.9%  126 | 0.405 |
| Communication requires involvement of next of kin* | 33.3% | 25.5% | 54.6% | 56.4% | 12.0% | 18.1% | 0.024 |
